# Comparative study of Dapagliflozin versus Glimepiride effect on insulin regulated aminopeptidase (IRAP) and interleukin-34 (IL-34) in patient with type 2 diabetes mellitus

**DOI:** 10.1038/s41598-023-33417-3

**Published:** 2023-04-18

**Authors:** Rania Zekry, Gamal A. Omran, Nashwa M. El-Gharbawy, Rehab H. Werida

**Affiliations:** 1 Department of Clinical Pharmacy & Pharmacy Practice, Faculty of Pharmacy, Damanhour University, Damanhour, 22514 Egypt; 2 Department of Biochemistry, Faculty of Pharmacy, Damanhour University, Damanhour, 22514 Egypt; 3 Diabetes and Endocrinology Unit, Department of Internal Medicine, Tanta University, Tanta, Egypt

**Keywords:** Endocrine system and metabolic diseases, Biomarkers, Diseases, Endocrinology

## Abstract

Type 2 diabetes mellitus (T2DM) is one of the most common diseases, that managed by several medications such as Glimepiride and Dapagliflozin. This study aims to compare the effects of Dapagliflozin versus Glimepiride on glycemic control, insulin resistance, and biomarkers as (extracellular domain of insulin regulated aminopeptidase) IRAPe, (interleukin-34) IL-34, and (N-terminal pro b-type natriuretic peptide) NT-proBNP. This study included 60 type 2 diabetic patients, who are randomized to receive either Glimepiride 4 mg/day (group 1) or Dapagliflozin 10 mg/day (group 2). Blood samples were collected at baseline and after 3 months of treatment for biochemical analysis. Additionally, HOMA-IR is calculated. After 3 months of receiving the intervention, there is no significant difference between the effects of Glimepiride and Dapagliflozin on FBG, PPBG, HbA1C%, fasting insulin, and HOMA-IR. The difference between both groups is significant for IL-34 (p = 0.002) and non-significant for IRAPe (p = 0.12) and NT-Pro BNP (p = 0.68). Both Glimepiride and Dapagliflozin significantly improve glycemic control, and HOMA-IR with no significant difference between them. Both drugs significantly improved the level of NT-proBNP. Dapagliflozin has a borderline significant effect on IRAPe but not IL-34, and Glimepiride has significant effect on IL-34 but not IRAPe.

**Clinical Trial Registration:** This trial was registered on clinicaltrial.gov (NCT04240171).

## Introduction

Diabetes mellitus is a chronic metabolic disease, in which there is a marked elevation in the blood glucose level. This elevation is associated with damaging effects on nerves, eyes, blood vessels, heart, and kidney. There are 2 main subtypes of diabetes: Type 1 Diabetes (T1DM), in which the pancreas is producing little or no insulin due to destruction of insulin-producing beta cells by autoimmune process. This type is known as insulin-dependent diabetes. Type 2 Diabetes (T2DM), in which the body become resistant to insulin causing functional deficit of insulin^1^. The management of patients with T2DM consists of diet and exercise along with other pharmacological therapies that target either insulin sensitivity or secretion. These pharmacological agents include: metformin, sulfonylureas, thiazolidinediones, α-glucosidase inhibitors, meglitinides, dipeptidyl peptidase-4 (DPP-4) inhibitors, Sodium Glucose cotransporter-2 (SGLT-2) inhibitors, Glucagon Like Peptide-1 (GLP-1) agonist, and selective amylin mimetics. The first line therapy is generally considered metformin^2^.

Glimepiride is a member of sulfonylureas family, which are used for the management of patients with T2DM. It works by increasing insulin secretion from pancreatic beta cells^3^. glimepiride is a modern second-generation sulfonylurea which is dosed once daily in the morning with meal. It has many advantages over older sulfonylureas including it is less likely to cause hypoglycemia, and it is not associated with cardiovascular harm^4^. Dapagliflozin is selective sodium glucose cotransporter-2 (SGLT-2) inhibitor, that is used in the management of patients with (T2DM). SGLT-2 is found in the proximal tubules of the nephron and is responsible for 90% of the resorption of glucose, so the inhibition of this cotransporter leads to glucose loss in the urine, resulting in decrease in blood glucose level and better glycemic control^5^. Dapagliflozin reduce the risk of hospitalization for heart failure in patients with type 2 diabetes and has been approved by FDA in 2020 for the treatment of heart failure because it reduces the composite outcome of cardiovascular death and worsening of heart failure in patients with heart failure with reduced ejection fraction (HFrEF)^6,7^. To our knowledge there are very few studies on the effects of glimepiride or dapagliflozin on insulin resistance, and there is no study that directly compare the effects of both drugs.

Homeostatic Model Assessment of Insulin Resistance (HOMA-IR) is a method for assessing insulin resistance from fasting insulin and fasting glucose concentration. The model has been proved to be a robust clinical and epidemiological tool. It has become one of the standard tools in the armamentarium of the clinical physiologist^8^.

One of the most important approaches of developing insulin resistance is the change in glucose transporters (GLUT)^9^. Normally, Insulin act to translocate GLUT-4 to the plasma membrane. An insulin regulated aminopeptidase (IRAP) is a cellular protein required for insulin-stimulated translocation of GLUT-4 to the plasma membrane^10^. In reaction to insulin, IRAP associates with and translocates to the plasma membrane with GLUT4 in skeletal muscle and adipose tissue, which are the two main glucose storage sites^11^. The extracellular region (IRAPs) of the protein is then cleaved and released into the bloodstream. IRAP transfer in reaction to insulin is significantly reduced in T2DM. IRAP, a protein linked to GLUT4 that is directly implicated in insulin-mediated glucose uptake^11^. Additionally, IRAPe appears to be a promising potential indicator for insulin resistance in T2DM population^11,12^.

Interleukin-34 (IL-34) is a cytokine that has been found to be expressed on adipocyte, and to be positively correlated with insulin resistance. IL-34 was found to significantly inhibits insulin-stimulated glucose transport in human differentiated adipocyte, which may explain its role in insulin resistance and making it a suitable marker to reflect insulin resistance status in T2DM patients^13,14^.

Natriuretic peptides (NP) are biomechanical biomarkers that are released from cardiac myocytes in response to inflammation, volume overload, hypoxia, and myocardial stretching. Natriuretic peptides action antagonizes the effects of renin angiotensin aldosterone system (RAAS), moreover, they reduce insulin resistance in the myocardial and skeletal muscles. Serum level of NP is used as a diagnostic tool for heart failure (HF) development and risk of cardiovascular death, as their serum level increase with the development of heart failure. NP have markedly used in the prediction of incident HF and total cardiovascular events in patients with type 2 diabetes^15^. The ADVANCE trial has shown that NT-proBNP levels are powerful prediction tools for the risk of HF, and death from cardiovascular events in patients with type 2 diabetes mellitus^16^. Although, until now, there is no recommendation by guidelines, NP have shown to be very powerful diagnostic and predictive tool for patients with type 2 diabetes mellitus to predict the risk of heart failure and other cardiovascular events. They can also be used for risk stratification in this population^15,17^.

### Aim of the work

This study aims to compare the effect of dapagliflozin versus glimepiride on glycemic control, HOMA-IR, and on markers like IRAPe, IL-34, and NT-proBNP, and to establish relationship between these parameters.

- Primary outcomes: change in the glycemic control and IRAPe.
- Secondary outcomes: change in the levels of IL-34 and NT-proBNP.

## Patients and methods

### Study design and participation

This is a 3-month prospective double blind parallel randomized study, that involve 60 type 2 diabetic patients of both sexes recruited from outpatient clinics of Diabetes and Endocrinology Unit, Department of Internal Medicine, Tanta University hospitals, Tanta, Egypt. The study took place from January 2021 to February 2022. All the procedures were done according to standards ethical guidelines and were approved by the research and ethics committee of Damanhour University (No. 1219PP20). The trial was registered on clinical trial.gov by (NCT04240171). All the study subjects have agreed to take part in the trial and provided a written informed consent. The study participants were asked to keep the usual dietary and activity habits throughout the study period. Patients were randomly assigned to either dapagliflozin group (n = 30) or glimepiride group (n = 30) in 1:1 ratio manner. All the patients were on metformin 1000 mg daily for at least a year before the beginning of the study. The dapagliflozin group received dapagliflozin 10 mg daily in addition to their usual dose of metformin, while the glimepiride group received glimepiride 4 mg daily in addition to their usual dose of metformin.

### Inclusion criteria

The selected patients were with uncontrolled type 2 diabetic with age ranging from 18 to 70 year and having glycated hemoglobin A1c% (HbA1c%) level ≥ 7. There were no limits to the duration of DM and gender.

### Exclusion criteria

Patients excluded from the study were those with type 1 diabetes, hepatic impairment, malignancy, heart failure, history of ischemic heart disease, planned surgical intervention, and both pregnant and nursing women, hypersensitivity to the study drugs, abnormal liver function, renal impairment (eGFR ≤ 60 ml/min) or history of bladder cancer.

### Demographic data and baseline evaluation

Complete medical examination was performed for each subject to evaluate their health status. Data on weight, height, sex, age, medical history, and medication were collected. BMI was calculated for each patient using the formula BMI = weight (kg)/height^2^ (m^2^).

### Study procedure and biomarker measurement

The blood samples were collected from each patient after overnight 12 hours (h) fasting period, a drop of blood was used to determine the fasting blood glucose using the glucometer. Patients were allowed to have breakfast, then after 2 h, the glucometer was used to determine the 2 h post prandial glucose. All these samples were collected at the beginning of the study and after 3 months of receiving the medication of interest. Blood samples were centrifuged at 2000–3000 rpm for 20 min, separated, collected in tubes, and stored at – 80 °C until biochemical analysis was performed. Fasting glucose, and 2 h. Post prandial glucose were determined using glucometer (Rightest, Bionime Corporation, Taiwan). Enzyme-Linked ImuunoSorbent Assay (ELISA) kits (Sunred biological technology Co., Ltd., Shanghai) were used in duplicate to determine fasting insulin (kit catalogue number: 201-12-0011, sensitivity: 0.352 mU/l, and assay range: 0.4–100 mU/l), glycated hemoglobin (Turbidimetric kit catalogue number: HBT1-100-0100, sensitivity: 1.4%, and assay range: 1.4%–14%), interleukin-34 (IL-34) (kit catalogue number: 201-12-0044, sensitivity: 4.336 ng/ml, and assay range: 5–1500 ng/ml), extracellular domain of insulin regulated aminopeptidase (IRAPe) (kit catalogue number: 201–12-4150, sensitivity: 0.236 ng/ml, and assay range: 0.25–72 ng/ml), and natriuretic peptides (NT-proBNP) (kit catalogue number: 201-12-1240, sensitivity: 1.117 pg/ml, and assay range: 2–360 pg/ml). Homeostatic Model Assessment of Insulin Resistance (HOMA-IR) was calculated from the equation:

$${\text{HOMA}} - {\text{IR }} = \, \left( {{\text{fasting insulin }}\left( {\upmu {\text{u}}/{\text{L}}} \right) \, \times {\text{ fasting glucose }}\left( {{\text{mg}}/{\text{dl}}} \right)} \right)/{4}0{5}.$$

### Follow up and adherence

All the study participants were followed up during the 3-month interval of the study, and all have confirmed adherence to their medications and their usual dietary and activity habits during the study.

### Sample size calculation

The required sample size was calculated using G*Power software version 3.1.0 (Institut für Experimentelle Psychologie, Heinrich Heine Universität, Dusseldorf, Germany). It was estimated that a total sample size of 60 patients would have a power of 95.4% to detect a medium to large effect size of 0.87 in the outcome measured.

### Statistical analysis

Statistical analysis of the data was performed, and box plots were created using IBM SPSS statistics software version 24 (IBM, Armonk, New York). Continuous variables are expressed as mean ± SD. Unpaired t-test was used to compare between the 2 groups, while paired t-test was used to detect the difference within the group between the beginning of the study and after 3 months. Categorical variables are expressed as numbers (percentage) and were compared using chi-square (X^2^) test. Correlation between variables were tested using Pearson correlation. The significance level was set at p < 0.05.

## Results

**Figure 1:**
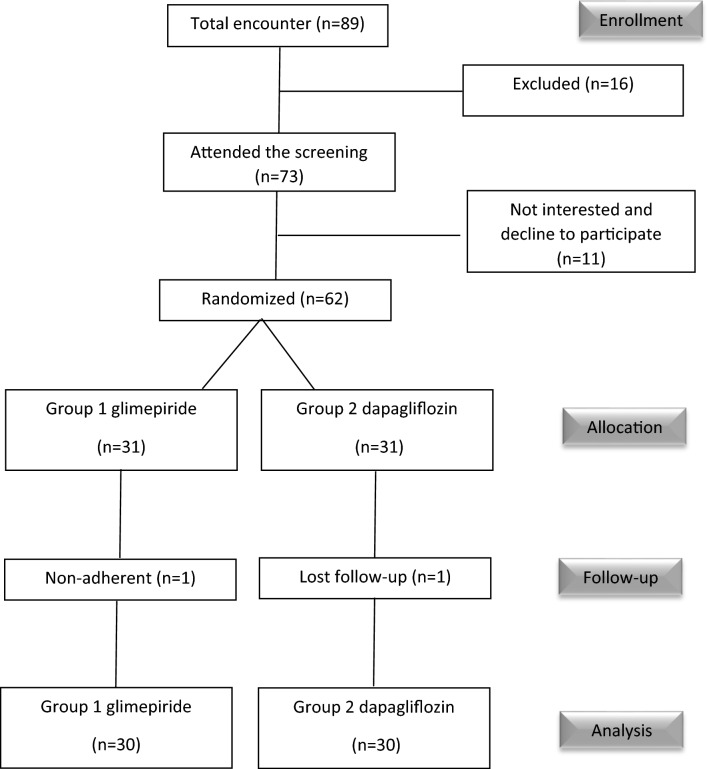
Flow-chart for study participants screening, enrollment, randomization, and follow-up.

Patients’ selection, randomization, and follow-up during the study are demonstrated in Fig. 1. Thirty subjects were enrolled in each group, glimepiride group and dapagliflozin group. Baseline characteristics of the participants are shown in Table 1.

**Table 1:** Baseline characteristics of patients participated in the study.

Parameters	Glimepiride group (n = 30)	Dapagliflozin group (n = 30)	p-value
Age	53.47 ± 7.65	55.07 ± 7.42	0.41*
Male/female	6 (20)/ 24 (80)	11 (36.7)/ 19 (63.3)	0.152^†^
Weight (kg)	93.43 ± 13.03	96.00 ± 10.31	0.40*
Height (cm)	166.67 ± 8.74	167.13 ± 7.59	0.83*
BMI (kg/m^2^)	33.92 ± 6.03	34.57 ± 4.84	0.65*
Hypertension	3 (10.0)	5 (16.7)	0.448^†^
ARB	14 (46.7)	13 (43.3)	0.795^†^
ACEI	3 (10.0)	5 (16.7)	0.448^†^

Data are presented as mean ± SD or number (percentage).

Data are analyzed using independent sample t-test or chi-square test as appropriate. The significance level was set at p < 0.05.

*BMI* body mass index, *ARB* angiotensin receptor blocker, *ACEI* angiotensin-converting enzyme inhibitor.

*Independent sample t-test.

^†^Chi-square (X^2^) test.

At base line, there was non-significant difference between the two studied groups as regarding demographic data and medical history as shown in Table 1; the 2 groups were matched for age [53.47 ± 7.65 vs. 55.07 ± 7.42 years; p = 0.41], sex ratio [M/F: 6/24 vs. 11/19; p = 0.152], weight [93.43 ± 13.03 vs. 96.00 ± 10.31 kg; p = 0.40], height [166.67 ± 8.74 vs. 167.13 ± 7.59 cm; p = 0.83], BMI [33.92 ± 6.03 vs. 34.57 ± 4.84 kg/m^2^; p = 0.65] in glimepiride group versus dapagliflozin group respectively. The medical history of the participants in glimepiride group versus dapagliflozin group were hypertension [3 vs. 5; p = 0.448], ARB use [14 vs. 13; p = 0.795], ACEI use [3 vs. 5; p = 0.448].

Biochemical data in glimepiride group versus dapagliflozin group were FBG [197.3 ± 67.08 vs. 212.1 ± 68.76 mg/dl; p = 0.40], PPBG [284.37 ± 56.47 vs. 306 ± 93.07 mg/dl; p = 0.28], HbA1c [9.32 ± 1.81 vs. 9.57 ± 1.87%: p = 0.60], Fasting insulin [19.98 ± 8.37 vs. 18.21 ± 11.74 µIU/ml; p = 0.50], HOMA-IR [9.86 ± 5.38 vs. 9.43 ± 5.93; p = 0.77], IRAPe [19.04 ± 7.03 vs. 18.42 ± 5.59 ng/ml; p = 0.71], IL-34 [462.37 ± 124.98 vs. 437.68 ± 136.02 pg. /ml; p = 0.47], and NT-ProBNP [93.29 ± 32.78 vs. 90.66 ± 43.35; p = 0.79] as shown in Table 2.

### Effects of glimepiride vs. dapagliflozin on fasting insulin and blood glucose level

Table 2 shows the studied variables at baseline and after 3-month follow up for both groups. The glimepiride group showed a significant reduction in fasting blood glucose (FBG) level (p = 0.002), 2-h post prandial blood glucose (PPBG) level (p < 0.0001), and glycated hemoglobin (HbA1C) (p < 0.0001), while fasting insulin showed no significant difference (p = 0.08). The dapagliflozin group also showed significant reduction in the level of the 3 variables, FBG level (p = 0.000001), PPBG level (p = 0.00016), and HbA1C level (p = 0.003), while fasting insulin showed no significant change (p = 0.11). There is no significant difference between the effects of glimepiride and dapagliflozin on FBG level (p = 0.164), PPBG level (p = 0.47), HbA1C level (p = 0.35), and fasting insulin level (p = 0.35).

**Table 2:** The outcomes of the study for both groups at baseline and 3 months after intervention.

Variables		Glimepiride group (n = 30)	Dapagliflozin group (n = 30)	P-value*
FBG (mg/dl)	Baseline	197.3 ± 67.08	212.1 ± 68.76	0.40
	After 3 months	173.1 ± 60.35	154.2 ± 41.92	0.164
	p-value^†^	0.002^‡^	0.000001^‡^	
PPBG (mg/dl)	Baseline	284.37 ± 56.47	306 ± 93.07	0.28
	After 3 months	248.8 ± 58.78	236.3 ± 74.83	0.47
	p-value^†^	< 0.0001^‡^	0.00016^‡^	
HbA1c (%)	Baseline	9.32 ± 1.81	9.57 ± 1.87	0.60
	After 3 months	8.38 ± 1.45	8.76 ± 1.67	0.35
	p-value^†^	< 0.0001^‡^	0.003^‡^	
Fasting insulin (µIU/ml)	Baseline	19.98 ± 8.37	18.21 ± 11.74	0.50
	After 3 months	18.36 ± 6.80	16.46 ± 8.76	0.35
	p-value^†^	0.08	0.11	
HOMA-IR	Baseline	9.86 ± 5.38	9.43 ± 5.93	0.77
	After 3 months	7.88 ± 3.53	6.47 ± 3.98	0.15
	p-value^†^	0.0018^‡^	< 0.0001^‡^	
IRAPe (ng/ml)	Baseline	19.04 ± 7.03	18.42 ± 5.59	0.71
	After 3 months	18.68 ± 4.39	20.75 ± 5.66	0.12
	p-value^†^	0.76	0.047^‡^	
IL-34 (ng/ml)	Baseline	462.37 ± 124.98	437.68 ± 136.02	0.47
	After 3 months	342.88 ± 87.14	451.43 ± 162.00	0.002^‡^
	p-value^†^	< 0.0001^‡^	0.53	
NT-Pro BNP (pg/ml)	Baseline	93.29 ± 32.78	90.66 ± 43.35	0.79
	After 3 months	71.32 ± 15.82	67.78 ± 43.55	0.68
	p-value^†^	0.001^‡^	0.01^‡^	

Data are presented as mean ± SD.

Data are analyzed using independent sample t-test or paired sample t-test as appropriate. The significance level was set at p < 0.05.

*FBG* fasting blood glucose, *PPBG* postprandial blood glucose, *HbA1C* glycated hemoglobin, *HOMA-IR* Homeostatic model assessment of insulin resistance, *IRAPe* extracellular domain of insulin regulated aminopeptidase, *IL-34* interleukin-34, *NT-proBNP N*-terminal pro b-type natriuretic peptide.

^†^Paired sample t-test.

*Independent sample t-test.

^‡^Statistically significant.

### Effects of glimepiride vs. dapagliflozin on insulin resistance as indicated by HOMA-IR

Table 2 shows that Glimepiride significantly lowered the HOMA-IR value (p = 0.0018), as well as the dapagliflozin (p < 0.0001), and there was no significant difference between the effect of both drugs on HOMA-IR (p = 0.15).

### Effects of glimepiride vs. dapagliflozin on biomarkers

**Figure 2:**
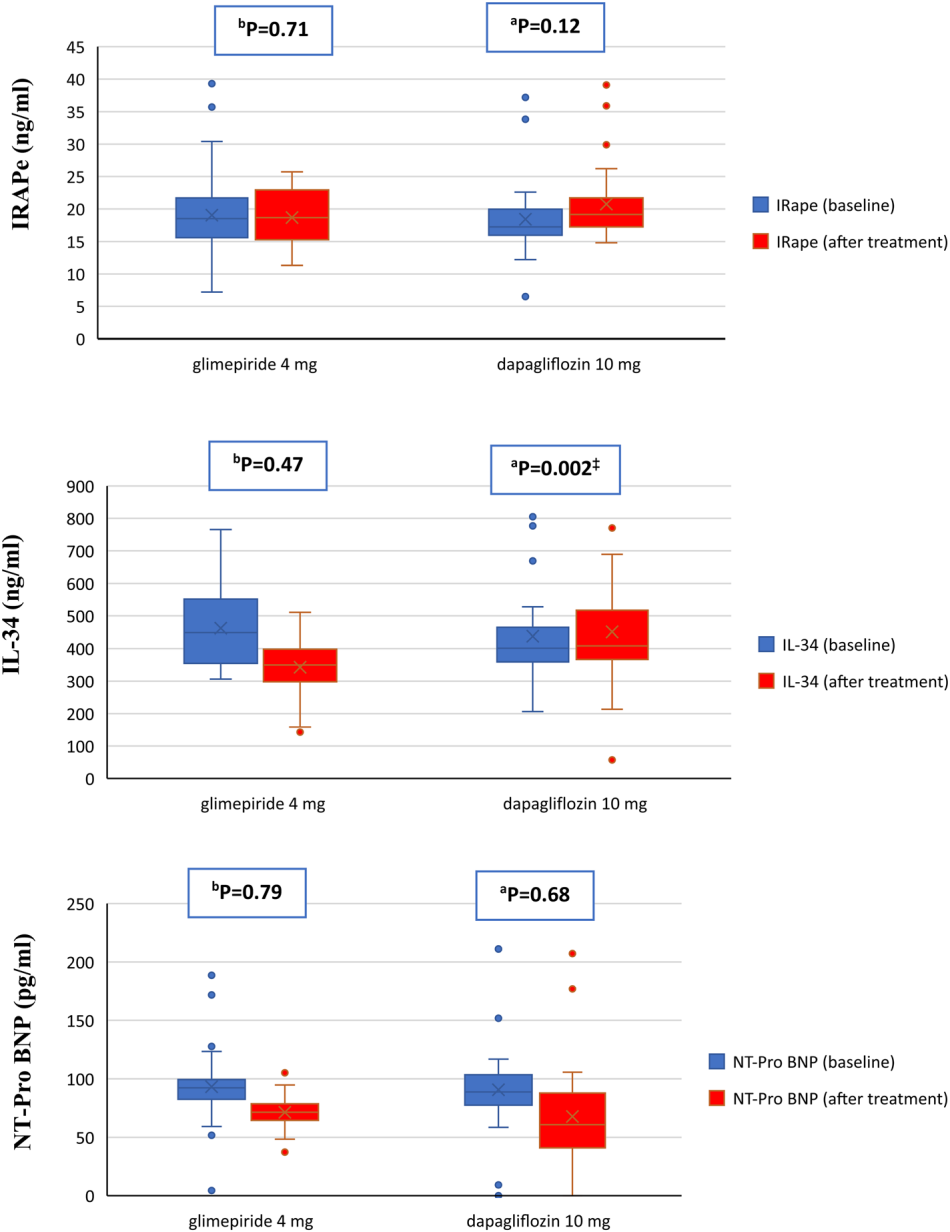
Changes of the measured biomarkers. *IRAPe* extracellular domain of insulin regulated aminopeptidase, *IL-34* interleukin-34, *NT-ProBNP N*-terminal pro b-type natriuretic peptide. Data analyzed by Independent sample t-test, ^b^P: baseline; ^a^P: after treatment.

Table 2 shows that glimepiride had no significant effect on IRAPe (p = 0.76), while it had significant effects on IL-34 (p < .0.0001), and NT-ProBNP (p = 0.001) compared to baseline. On the other hand, dapagliflozin group showed a borderline significant difference in IRAPe levels (p = 0.047), and significant difference in NT-ProBNP levels (p = 0.01), and there was no significant effect on IL-34 level (p = 0.53) compared to baseline. The difference between both groups is significant for IL-34 (p = 0.002) and non-significant for IRAPe (p = 0.12) and NT-Pro BNP (p = 0.68) as shown in Fig. 2 and Table 2.

Table 3 shows Pearson correlation analysis, NT-proBNP is positively associated with IRAPe and IL-34.

**Table 3:** Pearson correlation between NT-proBNP with IRAPe, and IL-34.

Parameter	NT-proBNP	
	r	P
IRAPe	0.419**	0.001
IL-34	0.404**	0.001

*IRAPe* extracellular domain of insulin regulated aminopeptidase, *IL-34* interleukin-34, *NT-proBNP* N-terminal pro b-type natriuretic peptide.

**Correlation is significant at the 0.01 level (2-tailed).

## Discussion

To our knowledge this is the first study that compares the effects of dapagliflozin and glimepiride on glycemic control, insulin resistance and different biomarkers as IRAPe, IL-34, and NT-pro BNP.

Our results show that both glimepiride and dapagliflozin have significantly improved the glycemic control in the study patients as they have significantly improved the levels of fasting and 2-h post prandial blood glucose level, and glycated hemoglobin. These findings are in accordance with the findings of Goldberg et al. that showed that glimepiride significantly reduce the levels of FBG, 2-H PPBG, and HbA1C (p < 0.001)^18^, and the finding of Ji et al. that showed that dapagliflozin significantly improved fasting and 2-h postprandial blood glucose levels as well as, glycated hemoglobin (p < 0.0001)^19^. We found that both drugs don't have significant effect on fasting insulin, unlike the findings of kabadi et al., which show that glimepiride significantly reduce fasting insulin (p < 0.01)^20^, but this may be due to the difference in the duration of our study and their study, however, Ramirez-Rodriguez et al. found that dapagliflozin don’t have significant effect on fasting insulin or insulin secretion in prediabetic patients^21^, which is in accordance with our findings, but our findings are in patients with type 2 diabetes. We have found that there is no significant difference between the effect of both drugs on FBG, 2-H PPBG, HbA1C, and fasting insulin.

HOMA-IR is widely used to assess insulin resistance using fasting glucose and fasting insulin^8^. We have found that both glimepiride and dapagliflozin have significantly reduced insulin resistance as indicated by HOMA-IR, these findings are in agreements with the findings of kabadi et al. which showed that glimepiride improved insulin sensitivity significantly^20^, and the findings of Merovci et al. that showed that the whole-body insulin sensitivity is improved by dapagliflozin^22^. We found that the effect of both drugs on insulin resistance is not significantly different.

IRAP plays an essential role in the translocation of GLUT-4 to plasma membrane, which plays an important role in the development of insulin resistance. When this translocation happens, the extracellular domain of IRAP (IRAPe) is released into the blood^10,11^. IRAPe is inversely proportional to insulin resistance, its level will increase when insulin resistance is reduced, and insulin sensitivity is improved. Our study has found that glimepiride do not have a significant effect on IRAPe, which is in accordance with the findings Elsheikh et al., that showed that glimepiride didn’t cause significant change in the level of IRAPe after 3 months of treatment^23^.

Our study observed that glimepiride decreased insulin resistance without having a significant effect on IRAPe level this may be attributed to HOMA-IR, that used as indicator for insulin resistance, which calculated based on fasting insulin and fasting glucose concentration. Glimepiride was found to stimulate glucose transport by affecting GLUT-1 and GLUT-4^24^. GLUT-1 is transmembrane protein allow passive transport of glucose^25^. Unlike GLUT-4, which is translocated to plasma membrane, and this translocation require IRAP and result in the cleavage of extracellular domain of IRAP (IRAPe) which increase the level of IRAPe in blood^10,11^. So, insulin resistance reflected by HOMA-IR may be improved by glimepiride without significant change of IRAPe.

Another possible explanation is that IRAPe may require more time to be improved in response to glimepiride or may require higher doses of glimepiride. And since our study is relatively short and the sample size is relatively small we cannot confirm the exact explanation.

We also found that dapagliflozin has significantly improved the level of IRAPe, but since the significance level is very close to the significance threshold and since our sample size is relatively small and the duration of the study is relatively short, this result needs to be confirmed with longer duration and larger sample size study. The difference between dapagliflozin and glimepiride effects on IRAPe is non-significant. We found that Glimepiride has significantly improved the level of IL-34, unlike dapagliflozin that do not have significant effect on its level. There is a significant difference between the effect of both drugs on IL-34. These findings may be explained as IL-34 is alternative ligand for colony stimulating factor-1 (CSF-1). IL-34 has been found to be involved in the inflammation and development of insulin resistance^13^. Glimepiride was found to have anti-inflammatory effects that is seen due to its effects on CSF^26^, while dapagliflozin's anti-inflammatory effect are mediated by NF-κB activation^27^.

NT-proBNP are biomarkers that are released from the heart in response to inflammation, hypoxia, or myocardial stretching. They have been proved to be a useful marker for the prediction of the development of heart failure in patients with type 2 diabetes^15^. We found that Both drugs have significantly improved the level of NT-proBNP, these findings are contradicting with Kusunose et al.^28^, whose findings showed that the level of NT-proBNP was elevated with glimepiride, and are in accordance with Jariwala et al.^29^, whose findings have showed that NT-proBNP level is significantly reduced and improved with dapagliflozin. There is no significant difference between the effect of both drugs on NT-proBNP.

## Conclusion

Both glimepiride or dapagliflozin significantly improved glycemic control, insulin resistance (as indicated by HOMA-IR), and NT-proBNP with no significant difference between them in type 2 diabetic patients. However, dapagliflozin has a borderline significant effect on IRAPe, and did not affect IL-34 significantly, while glimepiride has a significant effect on IL-34 but not IRAPe.

Since both drugs have improved the levels of NT-proBNP they can be recommended for diabetic patients who are at high risk for developing cardiovascular diseases (CVD). Both drugs have improved insulin sensitivity so they can be very useful for patients with high insulin resistance, with glimepiride more recommended for patients with high level of IL-34. Although, larger scale studies with longer follow-up duration are still needed to determine the benefits of both medications in diabetic patients.

### Study limitations

The main limitation of this study is relatively small sample size and short time of follow-up period. Hence, further large-scale studies seem necessary. Another limitation of our study is that it did not assess post prandial insulin resistance.

### Supplementary Information

Supplementary Information.

## Data Availability

All data generated or analysed during this study are included in this published article [and its Supplementary Information files].
