# Author Correction: Comparative study of Dapagliflozin versus Glimepiride effect on insulin regulated aminopeptidase (IRAP) and interleukin-34 (IL-34) in patient with type 2 diabetes mellitus

**DOI:** 10.1038/s41598-023-43905-1

**Published:** 2023-10-04

**Authors:** Rania Zekry, Gamal A. Omran, Nashwa M. El-Gharbawy, Rehab H. Werida

**Affiliations:** 1 Department of Clinical Pharmacy & Pharmacy Practice, Faculty of Pharmacy, Damanhour University, Damanhour, 22514 Egypt; 2 Department of Biochemistry, Faculty of Pharmacy, Damanhour University, Damanhour, 22514 Egypt; 3 Diabetes and Endocrinology Unit, Department of Internal Medicine, Tanta University, Tanta, Egypt

Correction to: *Scientific Reports* https://doi.org/10.1038/s41598-023-33417-3, published online 18 April 2023

The original version of this Article contained errors in the Reference List. The authors omitted the below References, which are now listed as Reference 28 and 29.

28. Kusunose K, Imai T, Tanaka A, Dohi K, Shiina K, Yamada T, Kida K, Eguchi K, Teragawa H, Takeishi Y, Ohte N, Yamada H, Sata M, Node k. effects of canagliflozin on NT-proBNP stratified by left ventricular diastolic function in patients with type 2 diabetes and chronic heart failure: a sub analysis of CANDLE trial. Cardiovascular diabetology. 2021;20,186. 10.1186/s12933-021-01380-w

29. Jariwala P, Jadhav K, Punjani A, Boorugu H, Mari AR. ADDition of DAPAgliflozin, Sodium-Glucose Cotransporter-2 Inhibitor to Angiotensin Receptor Blocker-Neprilysin Inhibitors Non-Responders in Patient with Refractory Heart Failure with Reduced Ejection Fraction (ADD DAPA trial). Indian Heart Journal. 2021;73(5):605–11. 10.1016/j.ihj.2021.07.005

As a result, in the Discussion section,

“We found that Both drugs have significantly improved the level of NT-proBNP, these findings are contradicting with Kusunose et al., whose findings showed that the level of NT-proBNP was elevated with glimepiride, and are in accordance with Jariwala et al., whose findings have showed that NT-proBNP level is significantly reduced and improved with dapagliflozin.”

now reads:

“We found that Both drugs have significantly improved the level of NT-proBNP, these findings are contradicting with Kusunose et al.^28^, whose findings showed that the level of NT-proBNP was elevated with glimepiride, and are in accordance with Jariwala et al.^29^, whose findings have showed that NT-proBNP level is significantly reduced and improved with dapagliflozin.”

The original Article has been corrected.

