# Supplementary material for: Comparative study of Dapagliflozin versus Glimepiride effect on insulin regulated aminopeptidase (IRAP) and interleukin-34 (IL-34) in patient with type 2 diabetes mellitus
Source: Sci Rep. 2023 Apr 18;13:6302. doi: 10.1038/s41598-023-33417-3 (PMC10113266; doi:10.1038/s41598-023-33417-3)
Supplement: Supplementary file 1 — Supplementary Information. [file 41598_2023_33417_MOESM1_ESM.pdf]

groupno	age(years)	sex	weight(kg)	height(cm)	metformin	ARB	ACEI	FG_Baselir	FG_aftertt
glimepiride	55.00	Female	120.00	170.00	Yes	Yes	Yes	218.00	190.00
glimepiride	40.00	Female	78.00	174.00	Yes	No	No	160.00	110.00
glimepiride	60.00	Female	105.00	157.00	Yes	Yes	No	205.00	180.00
glimepiride	41.00	Female	91.00	156.00	Yes	No	No	165.00	130.00
glimepiride	45.00	Female	93.00	162.00	Yes	No	No	94.00	92.00
glimepiride	62.00	Female	96.00	186.00	Yes	No	No	105.00	86.00
glimepiride	56.00	Female	108.00	173.00	Yes	Yes	No	175.00	144.00
glimepiride	65.00	Male	100.00	156.00	Yes	Yes	No	305.00	205.00
glimepiride	62.00	Female	85.00	165.00	Yes	No	No	135.00	120.00
glimepiride	61.00	Female	94.00	175.00	Yes	Yes	No	92.00	115.00
glimepiride	44.00	Male	73.00	167.00	Yes	No	No	158.00	130.00
glimepiride	56.00	Female	82.00	161.00	Yes	No	No	220.00	205.00
glimepiride	65.00	Female	92.00	188.00	Yes	No	No	311.00	290.00
glimepiride	60.00	Female	99.00	158.00	Yes	Yes	No	174.00	169.00
glimepiride	48.00	Female	89.00	170.00	Yes	No	No	325.00	288.00
glimepiride	52.00	Female	79.00	162.00	Yes	No	No	109.00	97.00
glimepiride	50.00	Female	110.00	178.00	Yes	Yes	Yes	138.00	116.00
glimepiride	61.00	Female	90.00	159.00	Yes	No	No	230.00	198.00
glimepiride	53.00	Male	102.00	168.00	Yes	Yes	No	176.00	153.00
glimepiride	49.00	Male	71.00	171.00	Yes	No	No	95.00	101.00
glimepiride	55.00	Female	95.00	160.00	Yes	Yes	No	184.00	209.00
glimepiride	51.00	Female	96.00	163.00	Yes	Yes	No	127.00	93.00
glimepiride	47.00	Female	115.00	169.00	Yes	Yes	Yes	151.00	123.00
glimepiride	61.00	Female	77.00	165.00	Yes	No	No	130.00	114.00
glimepiride	46.00	Female	93.00	172.00	Yes	No	No	208.00	196.00
glimepiride	54.00	Female	103.00	158.00	Yes	Yes	No	149.00	123.00
glimepiride	40.00	Male	90.00	176.00	Yes	Yes	No	108.00	91.00
glimepiride	45.00	Female	74.00	170.00	Yes	No	No	97.00	107.00
glimepiride	62.00	Male	117.00	156.00	Yes	Yes	No	219.00	195.00
glimepiride	58.00	Female	86.00	155.00	Yes	No	No	103.00	94.00
dapagliflozi	68.00	Female	93.00	167.00	Yes	No	No	164.00	145.00
dapagliflozi	44.00	Female	89.00	154.00	Yes	No	No	205.00	159.00
dapagliflozi	50.00	Male	91.00	162.00	Yes	Yes	No	182.00	142.00
dapagliflozi	43.00	Female	97.00	160.00	Yes	No	No	180.00	149.00
dapagliflozi	61.00	Male	79.00	172.00	Yes	No	No	210.00	130.00
dapagliflozi	38.00	Female	99.00	174.00	Yes	No	No	276.00	174.00
dapagliflozi	61.00	Female	120.00	169.00	Yes	No	No	350.00	155.00
dapagliflozi	61.00	Female	106.00	162.00	Yes	Yes	Yes	203.00	187.00
dapagliflozi	58.00	Female	110.00	160.00	Yes	Yes	Yes	183.00	172.00
dapagliflozi	64.00	Female	78.00	160.00	Yes	No	No	125.00	107.00
dapagliflozi	47.00	Female	95.00	159.00	Yes	Yes	No	95.00	94.00
dapagliflozi	61.00	Male	90.00	170.00	Yes	Yes	No	192.00	96.00
dapagliflozi	54.00	Male	103.00	167.00	Yes	No	No	249.00	121.00
dapagliflozi	59.00	Female	94.00	175.00	Yes	No	No	189.00	187.00
dapagliflozi	58.00	Female	110.00	155.00	Yes	Yes	No	325.00	240.00
dapagliflozi	61.00	Male	98.00	175.00	Yes	Yes	No	117.00	188.00
dapagliflozi	59.00	Male	75.00	165.00	Yes	No	No	346.00	173.00
dapagliflozi	45.00	Male	96.00	188.00	Yes	Yes	No	319.00	243.00
dapagliflozi	55.00	Male	80.00	170.00	Yes	No	No	120.00	70.00

dapagliflo:	60.00	Female	96.00	168.00	Yes	Yes	No	213.00	153.00
dapagliflo:	57.00	Female	100.00	180.00	Yes	Yes	Yes	186.00	158.00
dapagliflo:	58.00	Female	94.00	157.00	Yes	No	No	267.00	215.00
dapagliflo:	48.00	Male	92.00	171.00	Yes	No	No	211.00	155.00
dapagliflo:	50.00	Female	87.00	163.00	Yes	No	No	117.00	93.00
dapagliflo:	65.00	Female	113.00	169.00	Yes	Yes	Yes	212.00	150.00
dapagliflo:	55.00	Male	98.00	166.00	Yes	No	No	157.00	106.00
dapagliflo:	62.00	Male	105.00	170.00	Yes	Yes	Yes	307.00	202.00
dapagliflo:	52.00	Female	95.00	164.00	Yes	No	No	205.00	154.00
dapagliflo:	45.00	Female	96.00	177.00	Yes	No	No	220.00	132.00
dapagliflo:	53.00	Female	101.00	165.00	Yes	Yes	No	238.00	176.00

PP_baselir	PP_aftertt	HbA1c%_b	HbA1c%_a	F_insulin_l	F_insulin_a	HOMAIR_b	HOMAIR_a	QUICkI_ba	QUICkI_aft
227.00	218.00	8.30	7.50	23.10	18.20	12.43	8.54	0.27	0.28
264.00	207.00	9.10	8.40	32.50	24.60	12.84	6.68	0.27	0.29
384.00	340.00	10.70	10.20	37.80	27.50	19.13	12.22	0.27	0.27
224.00	192.00	8.10	7.30	21.70	19.60	8.84	6.29	0.28	0.29
158.00	128.00	5.20	5.10	5.19	9.30	1.20	2.11	0.37	0.34
240.00	207.00	6.90	6.60	25.80	21.70	6.69	4.61	0.29	0.31
327.00	291.00	10.90	10.30	14.40	12.90	6.22	4.59	0.29	0.31
387.00	325.00	14.60	10.10	31.90	21.30	24.02	10.78	0.25	0.27
266.00	198.00	8.80	7.50	11.80	6.72	3.93	1.99	0.31	0.34
248.00	228.00	12.10	10.10	15.20	31.40	3.45	8.92	0.32	0.28
275.00	264.00	7.80	7.20	15.80	16.50	6.16	5.30	0.29	0.30
265.00	225.00	9.50	8.70	27.60	28.30	14.99	14.32	0.26	0.27
380.00	367.00	12.20	11.60	20.50	16.60	15.74	11.89	0.26	0.27
261.00	226.00	8.60	7.90	24.00	23.80	10.31	9.93	0.28	0.28
408.00	384.00	11.80	10.50	14.90	18.10	11.96	12.87	0.27	0.27
200.00	163.00	7.40	6.60	30.00	25.40	8.07	6.08	0.28	0.29
243.00	209.00	8.60	7.90	21.00	19.80	7.16	5.67	0.29	0.30
300.00	276.00	11.00	10.20	30.40	25.50	17.26	12.47	0.26	0.27
283.00	244.00	10.80	9.90	13.60	12.40	5.91	4.68	0.30	0.31
174.00	145.00	7.30	6.30	7.70	6.80	1.81	1.70	0.35	0.35
317.00	285.00	9.60	8.90	16.60	15.20	7.54	7.84	0.29	0.29
230.00	192.00	7.90	6.80	19.50	19.00	6.11	4.36	0.29	0.31
249.00	210.00	8.40	7.20	20.30	21.90	7.57	6.65	0.29	0.29
269.00	249.00	9.20	8.40	18.20	17.90	5.84	5.04	0.30	0.30
233.00	176.00	8.30	7.50	24.80	22.10	12.74	10.70	0.27	0.27
252.00	230.00	9.40	8.70	11.00	13.40	4.05	4.07	0.31	0.31
167.00	140.00	7.00	6.70	22.40	21.30	5.97	4.79	0.30	0.30
124.00	112.00	9.90	9.00	12.70	11.50	3.04	3.04	0.32	0.32
264.00	243.00	10.00	9.20	31.00	26.30	16.76	12.66	0.26	0.27
236.00	198.00	7.90	6.80	27.00	24.70	6.87	5.73	0.29	0.30
260.00	225.00	12.20	10.80	8.87	10.20	3.59	3.65	0.32	0.32
237.00	233.00	10.10	9.60	12.10	10.30	6.12	4.04	0.29	0.31
305.00	179.00	7.50	6.40	68.30	45.60	30.69	15.99	0.24	0.26
236.00	167.00	10.40	10.80	15.60	17.60	6.93	6.48	0.29	0.29
302.00	274.00	10.90	9.70	20.90	13.90	10.84	4.46	0.27	0.31
423.00	330.00	11.70	10.50	12.60	6.43	8.59	2.76	0.28	0.33
416.00	178.00	13.90	8.20	15.30	22.10	13.22	8.46	0.27	0.28
340.00	315.00	8.80	10.10	23.50	23.70	11.78	10.99	0.27	0.27
215.00	270.00	8.50	7.30	41.10	37.60	18.57	15.97	0.26	0.26
189.00	198.00	6.60	7.50	7.36	9.85	2.27	2.60	0.34	0.33
112.00	116.00	6.40	6.50	7.63	13.30	1.79	3.09	0.35	0.32
244.00	150.00	7.60	9.10	15.70	13.30	7.44	3.15	0.29	0.32
440.00	200.00	9.20	9.30	5.86	5.63	3.60	1.68	0.32	0.35
240.00	216.00	9.10	8.90	16.80	7.83	7.84	3.62	0.29	0.32
566.00	305.00	11.20	8.70	11.70	21.20	9.39	12.56	0.28	0.27
221.00	239.00	7.70	6.11	19.40	20.90	5.60	9.70	0.30	0.28
415.00	190.00	9.50	9.30	7.13	4.81	6.09	2.05	0.29	0.34
400.00	491.00	10.60	10.50	24.50	16.10	19.30	9.66	0.26	0.28
256.00	214.00	5.80	5.80	12.60	7.16	3.73	1.24	0.31	0.37

307.00	241.00	7.60	6.70	19.56	18.40	10.29	6.95	0.28	0.29
257.00	231.00	8.70	7.50	17.50	19.60	8.04	7.65	0.28	0.29
301.00	309.00	10.50	10.60	22.30	16.26	14.70	8.63	0.26	0.28
306.00	160.00	11.60	12.00	20.26	21.80	10.56	8.34	0.28	0.28
197.00	155.00	6.70	5.80	13.96	14.10	4.03	3.24	0.31	0.32
308.00	315.00	9.80	9.90	21.60	20.10	11.31	7.44	0.27	0.29
302.00	240.00	7.50	6.80	16.70	13.80	6.47	3.61	0.29	0.32
355.00	232.00	11.40	9.00	14.26	15.30	10.81	7.63	0.27	0.29
311.00	163.00	10.60	8.60	15.52	11.90	7.86	4.52	0.29	0.31
304.00	236.00	8.60	6.20	13.60	12.80	7.39	4.17	0.29	0.31
415.00	317.00	12.80	11.00	24.00	22.26	14.10	9.67	0.27	0.28

iRape_bas	iRape_afte	IL34_basel	IL34_after	proBNP_ba	proBNP_afterttt
35.70	17.70	751.20	142.90	171.60	50.10
18.10	18.60	399.00	345.60	98.30	78.10
17.30	19.20	485.60	260.20	84.90	62.10
15.90	15.50	368.50	314.60	72.70	68.70
15.70	16.00	350.30	362.70	92.10	80.00
20.80	24.70	454.90	416.20	4.50	65.20
8.60	12.20	453.70	332.40	97.60	74.90
39.30	17.30	765.10	386.40	188.60	85.40
10.90	17.90	318.30	368.10	97.90	57.90
7.20	17.60	327.30	370.50	65.30	83.80
18.50	19.60	409.10	389.40	51.80	69.90
24.60	25.70	609.60	510.70	59.30	37.20
17.40	17.60	511.90	408.60	107.80	71.30
14.20	14.60	306.40	158.40	86.90	67.50
16.20	20.80	586.30	395.40	97.40	48.40
21.50	22.90	510.60	336.60	89.50	75.80
20.10	23.50	356.20	263.90	87.50	69.50
23.40	24.60	480.80	361.80	99.10	76.30
19.50	21.80	545.70	469.50	127.80	71.50
14.60	18.70	338.30	344.30	88.70	73.50
16.00	13.10	379.20	354.20	90.20	72.10
18.50	11.30	315.30	273.60	79.20	70.70
19.00	12.30	413.40	462.80	103.10	61.50
22.30	23.90	444.10	306.20	98.60	105.10
20.20	20.50	414.10	252.30	92.60	37.60
15.30	13.50	462.50	307.40	83.70	66.10
19.40	18.90	619.70	324.90	94.90	81.40
8.30	11.70	320.80	199.10	63.40	77.90
22.30	23.10	568.90	462.50	123.50	105.30
30.40	25.60	604.30	405.10	100.30	94.80
18.60	19.20	392.80	788.30	93.60	176.90
15.90	19.00	361.90	561.50	0.00	28.50
17.00	14.80	435.40	327.00	0.00	72.40
19.70	18.50	386.40	415.10	116.80	84.60
18.80	19.20	453.10	441.20	99.20	43.90
16.90	19.80	385.30	379.70	89.70	97.20
16.00	16.70	362.80	368.50	83.20	57.80
6.50	16.30	438.40	377.60	151.80	23.30
12.20	39.10	804.70	776.20	211.10	207.30
17.30	15.70	368.30	335.10	86.10	0.00
22.60	29.90	680.20	666.80	156.10	67.80
16.50	18.90	451.90	57.40	87.60	88.90
17.50	18.90	343.10	345.80	61.90	34.10
16.80	17.10	272.80	395.10	86.20	63.80
16.80	17.50	335.50	387.90	9.30	81.30
17.20	17.30	351.80	360.10	58.50	15.70
33.80	19.20	389.40	386.60	90.80	19.80
37.20	35.90	776.60	770.20	155.40	105.60
17.10	19.10	326.20	399.90	85.00	38.10

20.90	22.40	465.80	433.60	94.70	42.40
14.10	16.70	347.50	342.30	74.20	79.30
17.60	21.50	505.40	487.60	85.60	50.40
15.30	20.90	410.70	400.90	87.90	84.60
13.70	17.50	369.70	427.90	78.60	50.30
21.20	26.20	527.80	560.10	106.30	92.70
20.70	19.90	409.30	475.70	102.50	47.80
19.20	21.40	437.50	469.40	90.70	93.70
15.80	15.20	206.40	213.30	67.70	55.70
18.40	23.30	464.80	502.70	95.10	41.90
21.30	25.50	668.90	689.30	114.30	87.60
